# Supplementary material for: Testosterone Therapy for Late-Onset Hypogonadism: A Clinical, Biological, and Analytical Approach Using Compounded Testosterone 0.5–20% Topical Gels
Source: Pharmaceutics. 2024 May 6;16(5):621. doi: 10.3390/pharmaceutics16050621 (PMC11124925; doi:10.3390/pharmaceutics16050621)
Supplement: Supplementary file 1 [file pharmaceutics-16-00621-s001.zip › pharmaceutics-2895732-supplementary.pdf]

Supplementary Materials

Table S1. This table provides the description, acceptance criteria, and result from each parameter evaluated in the UPLC assay method validation. System suitability, linearity, accuracy, repeatability, intermediate precision, robustness, solution stability, and specificity of the method were evaluated as parts of the validation. Only when all parameters achieved the acceptable criteria, the method was considered accurate, precise, robust, and stability-indicating.

**Validation parameters**	**Description**	**Acceptance criteria**	**Results**	
**System suitability**	Ensures the chromatographic system is adequate for the assay analysis.	- Relative Standard Deviation (RSD) ≤ 1.0%<br>- Tailing factor ≤ 2.0<br>- Column efficiency ≥ 2000<br>- Resolution ≥ 2.0	RSD	0.3%
			Tailing factor	1.25
			Column efficiency	33456
			Resolution	n/a
**Linearity**	Verifies if the concentration-response relationship is directly proportional within the given range.	- $R^2 \geq 0.995$<br>- y-intercept ≤ 1.5% of target concentration<br>- Residuals ≤ 1.5% of target concentration	Regression line	6569.6 x + 1264.7
			R^2	1
			y-intercept	0.3%
			Maximum residual	0.59%
**Accuracy**	Establishes the closeness of the test results obtained by the analytical procedure to the expected value.	At 80%, 100%, and 120% of target concentration:<br>- 98.0% ≤ recovery ≤ 102.0%	*Testosterone 0.5% gel (placebo)*	
			Recovery at 80%	100.725%
			Recovery at 100%	101.213%
			Recovery at 120%	100.954%
			Testosterone 20% gel (placebo)	

			Recovery at 80%	100.595%
			Recovery at 100%	101.022%
			Recovery at 120%	100.845%
**Precision (repeatability)**	Determines the closeness of agreement among a series of measurements obtained from multiple sampling of the same homogeneous sample.	At 80%, 100%, and 120% from accuracy determination:<br>- RSD ≤ 2.0%	*Testosterone 0.5% gel (placebo)*	
			RSD at 80%	0.3%
			RSD at 100%	0.7%
			RSD at 120%	0.3%
			Testosterone 20% gel (placebo)	
			RSD at 80%	0.5%
			RSD at 100%	0.3%
			RSD at 120%	0.5%
**Precision (intermediate)**		From six determinations of standard solution at target concentration on two different days:<br>- RSD ≤ 5.0%	RSD from day 1, instrument 1	0.564%
			RSD from day 2, instrument 2	0.513%
**Robustness**	Establishes the capacity of the analytical method to remain unaffected by minor variations in the parameters.	With variations in column temperature, organic solvent content, and flow rate:<br>- RSD ≤ 2.0%<br>- Tailing factor ≤ 2.0<br>- Column efficiency ≥ 2000<br>- Resolution ≥ 2.0	*Conditions*	*Variations*
			Column temperature	± 2ºC
			Organic solvent content	± 5%
			Flow rate	± 1%
**Solution stability**	Determines the inter-day stability of the testing solutions.	From inter-day prepared standard solution and spiked	Stability (days)	4
			RSD from standard solution	0.475%

		placebo solution at target concentration:<br>- 97.0% ≤ recovery ≤ 103.0%<br>- RSD ≤ 2.0%	RSD from spiked placebo (testosterone 0.5% gel)	0.08%
			RSD from spiked placebo (testosterone 20% gel)	0.851%
**Specificity**	Forced degradation studies (thermal, acid, base and oxidation).	- No chromatogram interference<br>- 5-20% degradation in at least one stressed condition<br>- Resolution ≥ 2.0<br>- Purity flag: no	*Conditions*	*Thermal, acid, base, and oxidation*
			Degradation	Yes
			Interference	No
			Resolution	n/a
			Purity flag	No

n/a = not applicable

Table S2. The outcome of the stability study performed on testosterone 0.5% topical gel at room temperature was presented in this table. The stability study was conducted for 182 days. The physical characteristics (colour/appearance, odour, viscosity, and pH) and chemical attributes (assay and strength) were evaluated on days 0, 7, 14, 28, 42, 60, 90, 120, and 182. The preparation was stable throughout the duration of the study.

**Time Point**	**Physical Characterization**				**Chemical Characterization**		
	Colour/Appearance	*Odour*	*Viscosity (mPa.s)*	*pH*	*Assay (mg/g)*	*Strength (%)*	*Standard Deviation*
Day 0	Very faint beige, smooth	Characteristic	1044.7	5.75	5.014	100	0.871
Day 7	Very faint beige, smooth	Characteristic	1086.3	5.95	4.986	99.43	0.926
Day 14	Very faint beige, smooth	Characteristic	1081.7	5.69	4.925	98.23	0.801
Day 28	Very faint beige, smooth	Characteristic	1092.7	5.73	5.032	100.35	0.817
Day 42	Very faint beige, smooth	Characteristic	1091.7	5.98	5.084	101.40	0.498
Day 60	Very faint beige, smooth	Characteristic	1099.0	5.80	5.099	101.70	1.010
Day 90	Very faint beige, smooth	Characteristic	1146.0	5.88	5.085	101.42	0.878
Day 120	Very faint beige, smooth	Characteristic	1108.0	6.00	5.022	100.16	0.768
Day 182	Very faint beige, smooth	Characteristic	1059.0	5.78	5.058	100.87	0.784

Table S3. The outcome of the stability study performed on testosterone 0.5% topical gel at refrigerated temperature was presented in this table. The stability study was conducted for 182 days. The physical characteristics (colour/appearance, odour, viscosity, and pH) and chemical attributes (assay and strength) were evaluated on days 0, 7, 14, 28, 42, 60, 90, 120, and 182. The preparation was stable throughout the duration of the study.

Time Point	Physical Characterization				Chemical Characterization		
	Colour/Appearance	*Odour*	*Viscosity (mPa.s)*	*pH*	*Assay (mg/g)*	*Strength (%)*	*Standard Deviation*
Day 0	Very faint beige, smooth	Characteristic	1044.7	5.75	5.011	100	1.050
Day 7	Very faint beige, smooth	Characteristic	1110.0	5.63	4.968	99.13	0.991
Day 14	Very faint beige, smooth	Characteristic	1035.8	5.74	4.903	97.83	0.644
Day 28	Very faint beige, smooth	Characteristic	1103.3	5.79	5.009	99.96	0.824
Day 42	Very faint beige, smooth	Characteristic	1079.0	5.89	5.044	100.65	0.711
Day 60	Very faint beige, smooth	Characteristic	1086.3	5.77	5.094	101.65	0.847
Day 90	Very faint beige, smooth	Characteristic	1168.0	5.54	5.116	102.09	0.714
Day 123	Very faint beige, smooth *small separation appearance on top	Characteristic	1101.7	5.80	5.053	100.84	0.903
Day 182	Very faint beige, smooth *small separation appearance on top	Characteristic	1025.2	5.65	5.088	101.53	0.845

Table S4. The outcome of the stability study performed on testosterone 20% topical gel at room temperature was presented in this table. The stability study was conducted for 182 days. The physical characteristics (colour/appearance, odour, viscosity, and pH) and chemical attributes (assay and strength) were evaluated on days 0, 7, 14, 28, 42, 60, 90, 120, and 182. The preparation was stable throughout the duration of the study.

**Time Point**	**Physical Characterization**				**Chemical Characterization**		
	Colour/Appearance	*Odour*	*Viscosity (mPa.s)*	*pH*	*Assay (mg/g)*	*Strength (%)*	*Standard Deviation*
Day 0	White smooth	Characteristic	6595.0	5.89	207.443	100	0.921
Day 7	White smooth	Characteristic	4660.7	5.90	206.084	99.34	0.759
Day 14	White smooth	Characteristic	5300.0	5.87	204.484	98.57	0.963
Day 28	White smooth	Characteristic	4774.3	5.97	206.987	99.78	0.825
Day 42	White smooth	Characteristic	4546.0	5.93	211.339	101.88	1.038
Day 60	White smooth	Characteristic	4364.0	5.89	212.864	102.61	1.181
Day 90	White smooth	Characteristic	3452.0	5.73	208.850	100.68	0.934
Day 123	White smooth	Characteristic	3848.3	6.05	210.998	101.71	1.007
Day 182	White smooth	Characteristic	3738.0	5.67	210.955	101.69	1.015

Table S5. The outcome of the stability study performed on testosterone 20% topical gel at refrigerated temperature was presented in this table. The stability study was conducted for 182 days. The physical characteristics (colour/appearance, odour, viscosity, and pH) and chemical attributes (assay and strength) were evaluated on days 0, 7, 14, 28, 42, 60, 90, 120, and 182. The preparation was stable throughout the duration of the study.

**Time Point**	**Physical Characterization**				**Chemical Characterization**		
	Colour/Appearance	*Odour*	*Viscosity (mPa.s)*	*pH*	*Assay (mg/g)*	*Strength (%)*	*Standard Deviation*
Day 0	White smooth	Characteristic	6595.0	5.89	209.908	100	1.276
Day 7	White smooth	Characteristic	5257.3	5.76	207.605	98.90	1.244
Day 14	White smooth	Characteristic	5733.7	5.91	208.040	99.11	0.773
Day 28	White smooth	Characteristic	6088.3	5.97	209.924	100.01	0.509
Day 42	White smooth	Characteristic	5925.3	5.93	214.110	102.00	1.155
Day 60	White smooth	Characteristic	4932.7	6.05	213.147	101.54	1.182
Day 90	White smooth	Characteristic	4469.3	5.79	209.055	99.59	1.383
Day 123	White smooth	Characteristic	4548.0	5.93	209.733	99.92	0.912
Day 182	White smooth	Characteristic	3933.7	5.74	209.300	99.71	1.215

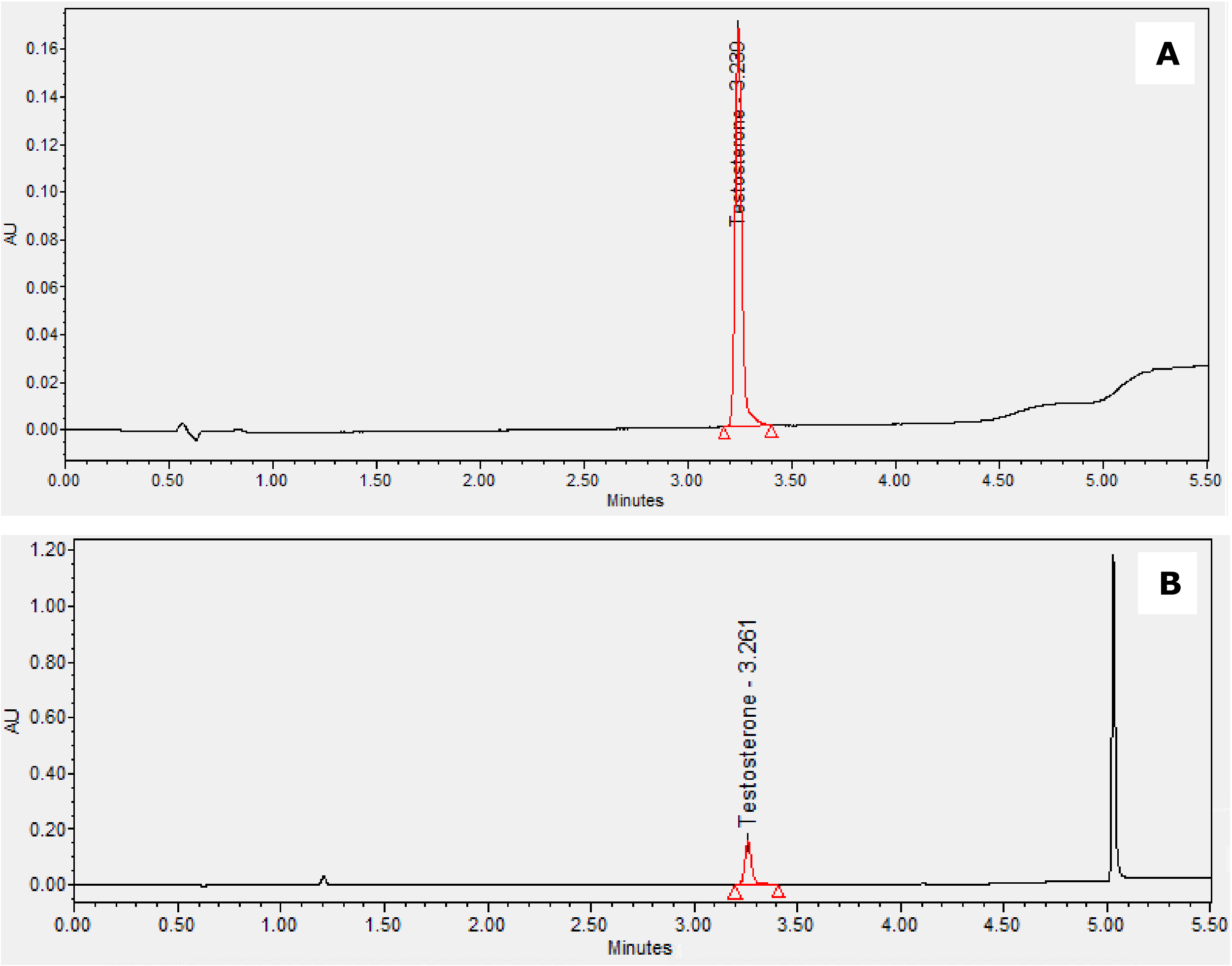

A
B
Testosterone - 3.230
Testosterone - 3.261
AU
Minutes
0.00
0.02
0.04
0.06
0.08
0.10
0.12
0.14
0.16
0.20
0.40
0.60
0.80
1.00
1.20
0.50
1.00
1.50
2.00
2.50
3.00
3.50
4.00
4.50
5.00
5.50

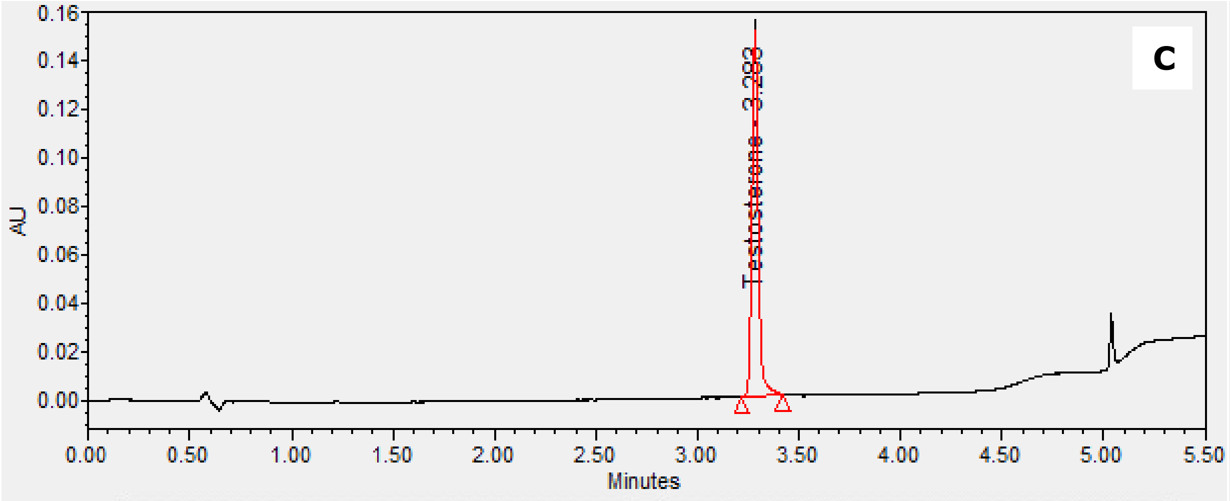

Figure S1. UPLC-PDA chromatograms (absorbance units vs. time) derived at 245 nm of testosterone in methanol (A) testosterone 0.5% topical gel (B); and testosterone 20% topical gel (C). The retention time of testosterone in this UPLC method was at about 3.25 minutes.
